# Heart rate increase results in case of positional venous entrapment

**DOI:** 10.1111/cpf.70041

**Published:** 2026-01-01

**Authors:** Quentin Petit, Simon Lecoq, Florian Congnard, Nathan Cronier, Pierre‐Yves de Müllenheim, Pierre Abraham, Bénédicte Noury‐Desvaux

**Affiliations:** ^1^ APCoSS, UCO‐IFEPSA, Institute of Physical Education and Sport Sciences Angers France; ^2^ Vascular Medicine, University Hospital Angers France; ^3^ Sports and Exercises Medicine, University Hospital Angers France; ^4^ UMR CNRS 1083 INSERM 6015 Angers France

**Keywords:** elevated arm stress test, heart rate, hemodynamics, pathophysiology, photoplethysmography, venous entrapment

## Abstract

**Introduction:**

Tachycardia has previously been reported as a possible sign of neurovascular entrapment during upper‐limb abduction and assumed to result from compression of the adrenergic nerve. However, this increase in heart rate could also be caused by a vascular factor, such as venous entrapment. The aim of this study was to determine whether heart rate increases specifically in the case of venous entrapment during upper‐limb dynamic mobilization tasks.

**Methods:**

One hundred and sixteen patients were asked to perform a provocative manoeuvre consisting of consecutive upper limb mobilizations by raising their arms to the “surrender” position (Su, 90° abduction) and then keeping their arms raised in front of the body (“prayer” position, Pra) prior to returning to the initial position (“End”). During this manoeuvre, simultaneous venous (V‐PPG) and arterial (A‐PPG) photoplethysmography (PPG) recordings were obtained. Participants were categorized by PPG recording analysis as having bilateral venous compression only (*V*‐group) or having no vascular compression (*C*‐group). All other responses (*n* = 75) were excluded. Heart rate responses in *V*‐group and *C*‐group were compared across arm positions using a linear mixed model.

**Results:**

*V*‐group (*n* = 17) showed a significantly higher heart rate during the ‘Su’ phase compared to the ‘Rest’ phase (+6.9 bpm, *p* < 0.001) and compared to the 24 patients of the *C*‐group (+4.9 bpm, *p* = 0.02).

**Conclusion:**

This study suggests that the cardiovascular response to dynamic provocative manoeuvres is found specifically in the presence of positional venous upper‐limb entrapment and likely results from decreased cardiac pre‐charge rather than from adrenergic nerve excitation.

## 1 INTRODUCTION

Thoracic outlet syndrome (TOS) of thoracic outlet compression (TOC), referring to a constellation of compressive problems that occur at the thoracic outlet, is rare. The structures usually involved in the thoracic outlet region are the first rib and the anterior scalene muscle, whose local imprint mainly affects the brachial plexus and subclavian vessels (Grunebach et al., 2015). While some authors estimated only 2.5 to 4 cases per 100 000 people per year in the general population (Illig & Rodriguez‐Zoppi, 2021), other studies have reported a broader incidence range, estimating between 3 and 80 cases per 1000 people (Li et al., 2021). There is a large number of scientific publications concerning TOS (>40 reviews in the last 2 years) and covering several fields such as physiopathology, etiology, epidemiology, non‐invasive diagnosis, invasive imaging, complications, and medical or surgical treatments (Abraham et al., 2023; Al‐Redouan et al., 2023; Daley et al., 2022; Hoexum et al., 2023; Perdikakis et al., 2023; Teijink et al., 2023). This may suggest that many questions remain unanswered about this puzzling medical issue. While most TOS are considered of neural origin due to the absence of arterial or venous complications, we will focus solely on hemodynamic responses on TOS suspected patients (Jones et al., 2019).

Clinical studies by Kaymak et al. (2004) and Özçakar et al. (2008) observed that preoperative tachycardia in some TOS patients resolved post‐surgery, hypothesizing that entrapment of the stellate ganglion was causing this rhythmic response. However, the precise mechanisms linking neurovascular compression, hemodynamic alterations, and heart rate modulation remain insufficiently understood.

Due to the conflict of space between the neurovascular bundle, osteo‐tendinous and muscular structures, the subclavian vein can become compressed, which may result in outflow impairment during upper limb abduction. Most individuals with positional neurovascular compression during abduction remain asymptomatic (TOC). Raaf (1955) observed that 60% of 75 normal (asymptomatic) participants experienced a disappearance of the radial pulse with shoulder retropulsion. Similarly, Gilroy and Meyer (1963) found that 69% of the arms of 40 normal participants exhibited a disappearance of the radial pulse or a supraclavicular murmur in various arm or shoulder positions. A more recent study by Chen et al. (2014) showed that in 100 upper limbs (50 normal participants), 60% had venous flow anomalies, including loss of flow phasicity, resulting in minimally continuous, or absent flow.

The observations in venous flow and pulsatility following possible positional occlusions during dynamic provocative manoeuvres (e.g., Roos test) (Chen et al., 2014; Gergoudis & Barnes, 1980; Gilroy & Meyer, 1963; Henni et al., 2019; Hersant et al., 2021; Raaf, 1955), may suggest that increases in heart rate (HR) are due to the vascular flow impairment (venous entrapment), with the right heart cavities receiving decreased inflow. While positional occlusions appear to influence cardiac responses, it remains unclear whether these heart rate changes primarily result from mechanical vascular impairment or autonomic dysregulation. Clarifying this relationship is essential for refining diagnostic criteria and optimizing therapeutic strategies for patients with TOS.

We have recently demonstrated the utility of venous photoplethysmography (V‐PPG) in evaluating venous return during dynamic manoeuvres in TOC (Hersant et al., 2022; Hersant et al., 2021; Hersant et al., 2021). The main aim of this study was to investigate heart rate changes during dynamic provocative manoeuvres in relation to the presence or absence of isolated venous outflow induced by positional compression with PPG. In this context, we hypothesized that, in cases of venous entrapment, the cardiovascular response may be blunted or even result in an increase in the heart rate due to decreased venous return if arterial inflow is preserved while venous outflow is impaired (entrapment of blood in the upper limbs).

## 2 METHODS

### 2.1 Study design and settings

This is a retrospective monocentric study that was submitted to the Ethics Committee of the University Hospital of Angers. It received a favorable opinion on March 12, 2024, under reference 2024‐053. In France, no formal consent was required. Patients were informed in writing, through their invitation letter, about the possibility of refusing the collection and use of their data for medical research purposes.

### 2.2 Study population

During consultations led by experienced practitioners, patient demographic data were collected, including age, gender, cardiovascular risk factors, personal history of thoracic or upper limb surgery, trauma to the upper limbs or cervical spine, current socio‐professional status, type and level of physical activity, and current treatments (both drug and non‐drug). Anthropometric measurements (weight, height, dominant arm) and resting blood pressure in both arms were also recorded.

**Figure 1:**
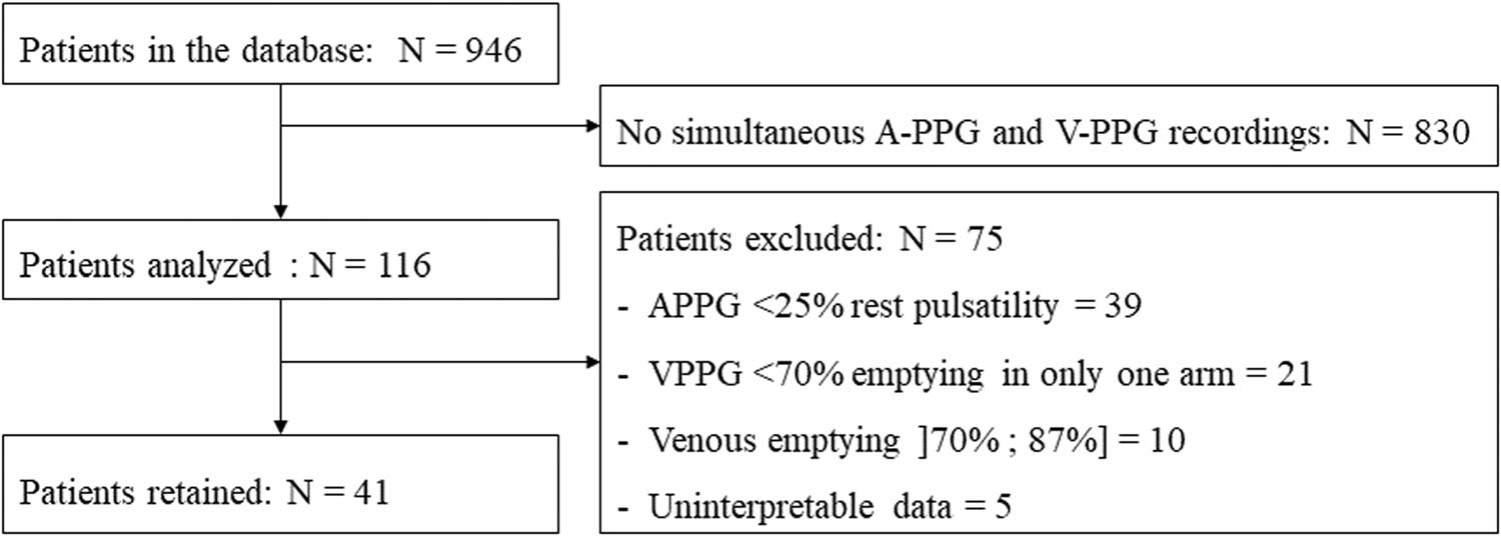
Flow chart depicting patient selection and exclusion criteria. A total of 946 patients were initially identified in the database. After excluding 830 patients due to the absence of simultaneous A‐PPG and V‐PPG recordings, 116 patients were included for analysis. Among them, 75 were excluded based on predefined criteria, including insufficient A‐PPG pulsatility, inadequate V‐PPG emptying, venous emptying greater than 70% and lower than 87%, or uninterpretable data. Ultimately, 41 patients were retained for the study.

The study population included patients referred to the vascular medicine department from the University Hospital of Angers for suspected TOS. Patients with TOS where chosen in reference to the two studies that have shown a tachycardia in this specific population (Kaymak et al., 2004; Özçakar et al., 2008). All patients were over 18 years old, with no upper age limit. Patients included exhibited symptoms of TOS and were undergoing PPG testing to assess potential vascular compressions as part of their diagnosis (Figure 1).

The inclusion criteria were as follows: (1) patients presenting with symptoms of TOS; (2) patients having performed dynamic manoeuvres to detect a TOC. The non‐inclusion criteria consisted of: (1) patients protected by law (pregnant women, parturient, nursing mothers); (2) individuals deprived of liberty by an administrative or judicial decision; (3) minors (<18 years old); (4) adults under legal protection or unable to consent; (5) patients who objected to the use of their data for research purposes or (6) any legal constraints.

The exclusion criteria were as follows: (1) patients with an arterial compression (remaining amplitude of arterial pulsatility <25% of resting pulsatility); (2) patients with a maximum venous emptying <70% during the manoeuvres in a single arm; (3) patients with a maximum venous emptying between 70% and 87%, in order to create two distinct groups either with clear venous obstruction (V‐PPG < 70% max) or clear normal response (V‐PPG > 87% max) (see Section 2.4); and (4) patients with uninterpretable recordings.

### 2.3 Experimental procedure

At the beginning of the procedure, patients completed the SF12 (Medical Outcomes Study 12‐item Short‐Form Health Survey) and the DASH (Disabilities of the Arm, Shoulder and Hand) questionnaires. The DASH score was calculated only if at least 90% of the first 30 questions were answered, requiring a minimum of 27 responses. Then, patients performed a modified version of the Roos test with simultaneous A‐PPG and V‐PPG measurements.

#### 2.3.1 Clinical positional manoeuvres

The Roos test is commonly used in the evaluation of patients with suspected TOS. It has been previously shown that upper limb elevation results in a physiological increase in Arterial pulse amplitude (A_pulse_) in normal subjects (Hickey et al., 2016). Therefore, we performed a 1‐min modified ‘candlestick‐prayer’ (“Ca+Pra”) clinical manoeuvre thereafter called ‘Su+Pra.’ Before initiating the manoeuvre, participants were asked to maintain their arms in a relaxed position along the sides of the body for at least 1 min, allowing full venous filling and hemodynamic stabilization prior to data recording. Following this period, the data acquisition process (30 s) was initiated to record the physiological responses during the manoeuvre and stopped at least 1 min after the end of the provocative manoeuvre. Patients in sitting position were asked to stay in the previous calibration position (arms resting against their sides) for an additional 5 s of recording (Rest). Then, patients were asked to start an upper limb movement according to modified version of the Roos test (Hersant et al., 2021) during which the surrender position used for the Roos tests (Su) was maintained for 30 s (without opening and closing of hands to avoid movement artifacts on the A‐PPG recordings). After this stage, patients were invited to move their arms to the prayer position (Pra), without lowering the hands and with elbows in front of them, for 15 s. In the ‘Pra’ position, elbows and hands are at the same level relative to the heart level as for the ‘Su’ position. The purpose of the ‘Pra’ position is to open the costoclavicular angle and achieve arm elevation without vascular compression. After the ‘Pra’ position, the upper limbs were lowered and immobilized for 10 s in this position (End). Due to PPG software limitations, recordings could only be obtained for a maximum duration of 60 s during this manoeuvre.

#### 2.3.2 Photoplethysmography recordings

V‐PPG was measured on each forearm (2–3 cm distal from the elbow crease) at a sampling rate of 4 Hz (Sino‐K, Shenzhen, CN). The system allowed for the bilateral recording of low‐pass filtered plethysmographic signal and recorded 5 s at rest, used as zero volume (forearm complete filling), while the arms are alongside the torso in sitting position. Increases from zero denotes forearm emptying and a decrease denotes forearm filling. Values are recorded in arbitrary units. Elevating the upper limb in the surrender position is expected to result in venous emptying that persists as long as the arms remain about heart level. These recordings make it possible to know if there is an isolated venous compression if the artery is not occluded resulting in a filling of the forearm during the manoeuvres.

Arterial pulse photoplethysmography (A‐PPG) was performed simultaneously on the second finger of both hands using SpO_2_ (pulse oxygen saturation) soft‐tip adult finger sensors at a rate of 125 Hz (Sino‐K, Shenzhen, CN) and at the ear lobe. Recording was initiated at least 30 s before the start of the positional manoeuvre. Each recording enabled the detection of the A‐PPG signal during each cardiac cycle. A_pulse_ was determined as the difference between the maximum and minimum A‐PPG values over all 0.008 s intervals. Normally, two signals can be extracted from the raw plethysmography signal. One signal corresponds to the small rhythmic changes resulting from pulsatility, and can be separated from the raw signal by a high‐pass filter (Hersant et al., 2021). The other signal corresponds to slow amplitude changes assuming to result from venous volume changes. In theory both could be extracted from the same recording but venous photoplethysmography (V‐PPG) is optimally performed at the elbow level (Hersant et al., 2022) while arterial pulsatility is better detected at the fingertip level (Hersant et al., 2021).

Moving averaging on pulse amplitude was applied over each series of 10 consecutive points (Hersant et al., 2021). At the fingertip level, a transient artifact peak was systematically observed at upper limb elevation and lowering. Then, A_pulse_ at rest and A_pulse_ during the ‘Su’ position were calculated respectively between 20 and 5 s before and between 5 and 20 s after the manoeuvre was started to avoid the movement artifact influencing recorded results. This was allowed by the calibration period included within the protocol. Since arm elevation results in a physiological increase in A_pulse_ in normal subjects (Hickey et al., 2015, 2016), we normalized A_pulse_ as a percentage of the A_pulse_ observed during the “Pra” position (between 35 and 40 s after the beginning of the provocative manoeuvre). A_pulse_ changes were expressed as the comparison between A_pulse_ during the ‘Su’ position and A_pulse_ observed at rest, and were expressed as a percentage of resting pulsatility.

### 2.4 Classification of patients

Patients with V‐PPG recordings confirming the presence of bilateral pure venous compression (remaining amplitude of arterial pulsatility >25% of resting values and maximum venous emptying <70% during the “Su‐Pra” manoeuvers) were included in the venous group (*V*‐group).

Patients were included in the control group (*C*‐group) only in case of complete absence of vascular compression (neither venous nor arterial) in both arms (remaining amplitude of arterial pulsatility >25% of resting values and maximum venous emptying >87% during the “Su‐Pra” manoeuvres). These criteria were taken from studies by Gergoudis et Barnes; Geven et al. and Hersant et al. (Gergoudis & Barnes, 1980; Geven et al., 2006; Hersant et al., 2022a). We used these restrictive criteria in order to exclude all patients who might have a moderate unilateral or bilateral non‐significant arterial or venous compression.

### 2.5 Heart rate extraction

Heart rate was derived from A‐PPG recordings obtained at the earlobe, using AcqKnowledge® software (version 3.9.2, BIOPAC Systems, Inc., USA) (Kremer et al., 2008). The A‐PPG signal was processed with a high‐pass filter, utilizing the Infinite Impulse Response (IIR) command to ensure signal integrity. HR values were then extracted and expressed in beats per minute (bpm).

Occasionally, HR extractions using AcqKnowledge® contained artifacts resulting from hardware anomalies or missing data. In such cases, adjustments were made, and missing values were interpolated from adjacent data points to preserve data continuity. HR data were recorded over a 60 s period, with values exported at 0.4 s intervals for further analysis.

### 2.6 Data analysis

Data analyses were performed using R programming language (version 4.4.1) and the RStudio environment (version 2024.12.0 + 467).

To enhance the interpretability of HR changes over time, HR data were normalized for each patient by subtracting the median of the resting HR values from the HR values recorded during the manoeuvre phases. This normalization facilitated the visualization of between‐group differences. The median was chosen over the mean due to its robustness against outliers, which are common in physiological recordings during dynamic manoeuvres. For each patient, the median of normalized HR was computed for each manoeuvre phase. For visualization purposes, we also computed the median and the interquartile range of normalized HR for each time interval for both *C*‐group and *V*‐group. These data were smoothed using a 6 s window width. The distributions of median normalized HR for both groups and each manoeuvre phase were presented through raincloud plots.

To investigate the effect of venous compression on normalized HR across manoeuvre phases, a linear mixed model was built with normalized HR as the response variable, group, manoeuvre phase and the interaction as fixed effects, and a random intercept for participants. This kind of model accounts for the hierarchical structure of the data when evaluating the effects of various factors and interactions on the dependent variable while adjusting for individual‐level variability. Model quality was verified by analyzing normality and homogeneity of residuals.

From the model, the *emmeans* package (Lenth et al., 2024) was used to conduct pairwise comparisons of estimated marginal means (EMMs) of median normalized HR between groups (venous compression vs. no venous compression) across the manoeuvre phases, and between phases across groups, while using the Holm method to control the family‐wise error rate. The choice of analyzing EMMs of medians, rather than means of raw data, was made to combine the robustness of median values against outliers with the adjustment capabilities of mixed models. This ensures that observed differences accurately reflect true group and phase effects rather than being influenced by extreme values or unbalanced repeated measures. An alpha level ≤5% was considered to claim statistical significance.

## 3 RESULTS

### 3.1 Population description

Forty‐one patients were included in the study, with a mean ± SD age of 42 years old (±10 years old). The gender distribution was 75.6% female and 24.4% male. Mean arterial pressure measurements at rest were 130/79 mmHg ( ± 15/10 mmHg) for the right arm and 132/79 mmHg ( ± 16/10 mmHg) for the left arm.

The SF‐12 survey yielded scores of 35.6 (±6.24) for physical quality of life and 37.4 (±7.04) for mental and social quality of life, both below the threshold of 50, indicating a below‐average quality of life (Gandek et al., 1998). The average DASH score was 50.2 (±19.1), indicating an average disability of 50% in daily life (Atroshi et al., 2000), with a non‐retention rate of 14.6%.

All 41 patients underwent exploratory examinations such as Doppler ultrasound (78.0%) and phlebography (53.7%). Among the patients who underwent Doppler ultrasound, 51.2% were diagnosed with abnormalities, including thrombosis, aneurysm, or other vascular pathologies. For the 22 patients who underwent dynamic phlebography, 39.0% had results supportive of TOS. Finally, all patients underwent venous and arterial PPG. Based on the classification criteria, 24 patients were assigned to the *C*‐group and 17 to the *V*‐group. Their resting HR values were respectively 89.8 ( ± 15.1) and 88.4 ( ± 10.6) bpm.

### 3.2 Heart rate changes

**Figure 2:**
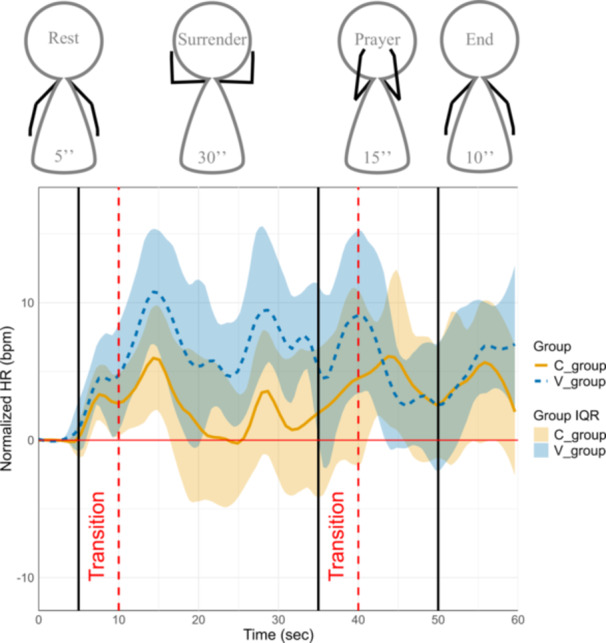
Evolution of median IQR (interquartile range) normalized heart rate over time and phases in both groups. Median (solid brown line and dashed blue line) and interquartile range (shaded area between Q1 and Q3) of normalized heart rate are shown for both groups: *C*‐group (solid brown) and *V*‐group (dashed blue). Values are presented relative to baseline, over time and over phases. Interquartile ranges are presented for both groups. Vertical black lines indicate the limits between the different manoeuvre phases, while vertical dashed red lines denote transition periods excluded from analysis to minimize motion artifacts.

Heart Rate changes across the manoeuvre phases are depicted in Figure 2. Statistical analysis showed that only the ‘Su’ phase demonstrated a significant between‐group difference in normalized HR (*V*‐group > *C*‐group, *p* = 0.02), and that only the venous group exhibited a significant HR increase from Rest to Su (*p* < 0.001), whereas no other phase showed significant changes within or between groups. During the transition to the ‘Su’ phase, both groups exhibited a transient HR increase, with peaks of approximately 11 and 6 bpm above baseline for the *V*‐ and *C*‐groups, respectively. Subsequently, the *C*‐group's HR decreased to values near baseline, whereas the *V*‐group maintained HR variations over 6 bpm above baseline.

During the transition to the ‘Pra’ phase, both groups experienced another transient HR increase, reaching levels similar to those observed during the ‘Su’ phase transition. This was followed by a gradual HR reduction during the ‘Pra’ phase, with both groups returning to values approximately ±3 bpm above their resting HR (end of the “Pra” phase).

In the ‘End’ phase, both groups demonstrated another HR increase. For the *C*‐group, HR rose by approximately 2 bpm before dropping back by 2 bpm. In contrast, the *V*‐group showed a distinct trend: an initial increase of approximately 4 bpm at the start of the phase, followed by a maintenance of the HR by the end of the manoeuvre (7 bpm above baseline). The interquartile range (IQR) showed exactly the same trends for their respective groups.

**Figure 3:**
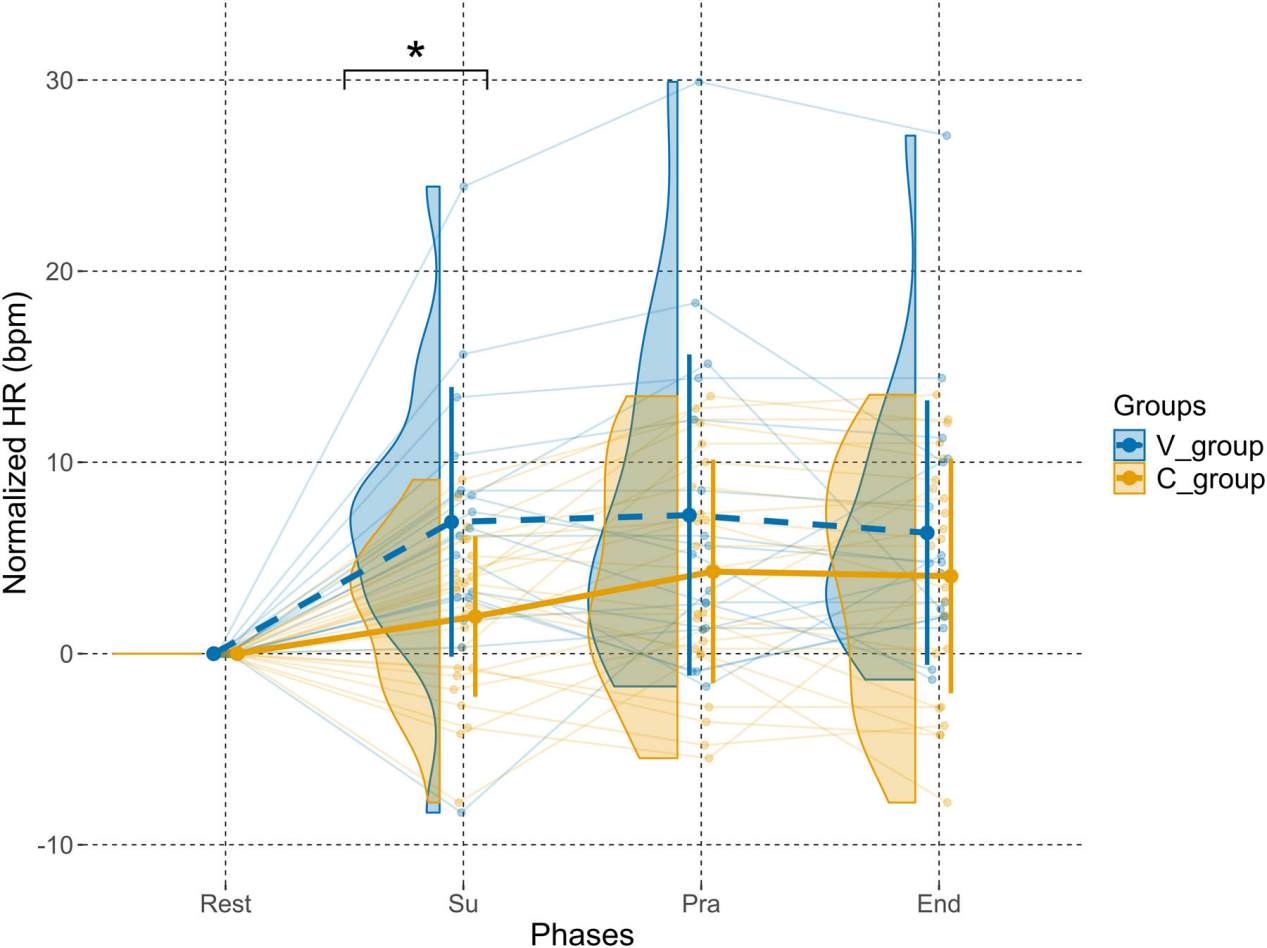
Changes in normalized heart rate (HR) across phases in both groups. Violin plots represent the distribution of normalized HR values for each phase in the venous group (*V*‐group, blue) and the control group (*C*‐group, brown). Individual data points are connected by light lines. Thick solid lines indicate group means, with vertical error bars showing ±1 standard deviation (SD). Values are expressed as changes relative to baseline (Rest). The asterisk (*) denotes a significant difference between groups (*p* < 0.05).

Figure 3 and Table 1 highlight the mean of the mean normalized HR differences between the *V*‐group and the *C*‐group during all phases. For the ‘Rest’ phase, values were null due to the manipulation of the data to normalize every value to their resting value.

**Table 1:** Absolute and normalized HR values in bpm depending on the group and the phases.

		Rest	Su	Pra	End
*C*‐group	Absolute HR (SD)	84.9 (13.4)	86.9 (13.7)	89.2 (15.0)	89.0 (14.9)
	Normalized HR (SD)	0.0 (0.0)	1.9 (4.2)	4.3 (5.8)	4.1 (6.1)
*V*‐group	Absolute HR (SD)	81.5 (10.1)	88.4 (7.1)	88.7 (9.2)	87.8 (10.7)
	Normalized HR (SD)	0.0 (0.0)	6.9 (7.1)	7.2 (8.4)	6.3 (6.9)

During the ‘Su’ phase, the mean normalized HR increased by 6.9 bpm ( ± 7.1) in the *V*‐group compared to the ‘Rest’ phase, while the *C*‐group exhibited a smaller increase of 1.9 bpm ( ± 4.2), resulting in a between‐group difference of 5 bpm.

During the “Pra” phase, the difference between the *V*‐ and *C*‐groups was attenuated. The mean normalized HR in the *V*‐group remained slightly higher than in the *C*‐group, with values of 7.2 bpm ( ± 8.4) and 4.3 bpm ( ± 5.8), respectively, corresponding to a reduced intergroup difference of 2.9 bpm.

A slight difference was also observed during the ‘End’ phase, with the *V*‐group showing a mean HR of 4.1 bpm ( ± 6.1) and the *C*‐group reaching 6.3 bpm ( ± 6.9), leading to a between‐group difference of 3.2 bpm. The mean HR variation during the ‘End’ phase was comparable to that observed in the ‘Pra’ phase.

Table 2 assessed differences between *V*‐group and *C*‐group across the mobilization phases and assessed differences between phases across groups. During the ‘Su’ phase, median normalized HR was higher than during the ‘Rest’ phase for both the *V*‐group ( + 6.9 bpm) and the *C*‐group ( + 1.9 bpm). It indicates a significant difference of 4.94 bpm (*t*(89.3) = 2.83, *p* = 0.02) between both groups.

**Table 2:** Between‐group comparisons of the means of median normalized HR across the manoeuvre phases.

Contrast	Reference	Estimate	Standard error	df	t. ratio	*p* value	Significance
*V*‐group–*C*‐group	Rest	0.00	1.75	89.3	0.00	1.00	
*V*‐group–*C*‐group	Su	4.94	1.75	89.3	2.83	0.02	*
*V*‐group–*C*‐group	Pra	2.95	1.75	89.3	1.69	0.28	
*V*‐group–*C*‐group	End	2.27	1.75	89.3	1.30	0.39	
Su – Rest	*V*‐group	6.89	1.34	117	5.15	<0.0001	***
Pra – Rest	*V*‐group	7.25	1.34	117	5.42	<0.0001	***
End – Rest	*V*‐group	6.33	1.34	117	4.73	0.0001	***
Pra – Su	*V*‐group	0.36	1.34	117	0.27	1.00	
End – Su	*V*‐group	−0.56	1.34	117	−0.42	1.00	
End – Pra	*V*‐group	−0.92	1.34	117	−0.69	1.00	
Su – Rest	*C*‐group	1.95	1.34	117	1.73	0.43	
Pra – Rest	*C*‐group	4.30	1.34	117	3.81	0.002	***
End – Rest	*C*‐group	4.10	1.34	117	3.60	0.004	***
Pra – Su	*C*‐group	2.35	1.34	117	2.10	0.27	
End – Su	*C*‐group	2.11	1.34	117	1.87	0.38	
End – Pra	*C*‐group	−0.24	1.34	117	−0.21	1.00	

*Note*: *p* values are adjusted using the Holm method.

*p < 0.05; ***p < 0.001.

In the ‘Pra’ phase, we observed no difference between *V*‐ and *C*‐groups ( + 2.95, *t*(89.3) = 1.69, *p* > 0.05). No between‐group difference was observed during the ‘End’ phase ( + 2.27, *t*(89.3) = 1.30, *p* > 0.05).

Then, compared to the ‘Rest’ phase, the ‘Su’ ( + 6.89, *t*(117.0) = 5.15, *p* < 0.001) phase showed a significantly increased chronotropic response. However, over the course of the 60‐second manoeuvres, no significant differences were observed between ‘Su’ and ‘Pra’ phases ( + 0.36, *t*(117.0) = 0.27, *p* > 0.05), and between ‘Pra’ and ‘End’ phases (−0.92, *t*(117.0) = −0.69, *p* > 0.05). In the *C*‐group, no significant differences were identified between ‘Rest’ and ‘Su’ phases, ‘Su’ and ‘Pra’ phases and ‘Pra’ and ‘End’ phases (all *p* > 0.05).

## 4 DISCUSSION

To the best of our knowledge, there is a paucity of comprehensive studies quantifying HR changes during upper limb mobilization in TOS subjects. Clinical observations of tachycardia in some subjects with TOS have been noted anecdotally but have not been systematically evaluated. Recent studies by Abraham et al. (2020), Henni et al. (2019), Hersant et al. (2022) and Hersant, Ramondou, Hersant et al., (2021) have employed analogous protocols involving V‐PPG and A‐PPG, enabling the extraction and analysis of HR, thereby demonstrating the feasibility of a novel approach to quantifying cardiovascular response among TOS patients. The objective of this study was to assess the cardiovascular response, particularly HR response, in patients with isolated venous outflow impairment without arterial inflow restriction due to TOC, in comparison to patients exhibiting no observable venous or arterial inflow impairments during dynamic provocative manoeuvres (“Su + Pra”), showing a relative higher HR in the presence vs the absence of venous entrapment.

Our findings reveal interesting insights into HR dynamics during provocative manoeuvres. First, in both groups, the start of each phase led to an initial increase of HR (first transition phase in Figure 2), probably due to upper limb mobilization against gravity, which provokes a compensatory HR increase, as observed by Couser et al., 1992. Then, the difference in cardiovascular response observed between groups during the ‘Su’ phase could reflect the distinctive biomechanical demands of the manoeuvre. With the arm in abduction and the elbow flexed, venous entrapment occurs at the thoracic outlet, resulting in compromised venous return. This restriction is supported by V‐PPG measurements, which indicate venous pooling in the arm due to limited venous outflow, as observed by Hersant, Ramondou, Chavignier et al. (2021). As a result, venous stasis could lead to reduced venous return to the heart, decreasing cardiac preload. Consequently, in the ‘Su’ phase, patients with venous TOS exhibit a compensatory increase in HR compared to the ‘Rest’ phase ( + 6.9 bpm, *p* < 0.001). A significant difference was also observed between both groups during this phase ( + 4.9 bpm, *p* = 0.02 for *V*‐group compared to *C*‐group). This slight but significantly higher cardiac response in patients with venous entrapment could highlight the potential additional cardiovascular strain caused by venous flow obstruction.

Such a cardiac response during arm elevation and abduction has already been reported by Kaymak et al. (2004). A 22‐year‐old patient with a neurogenic TOS diagnosis was evaluated with a Holter monitor during the performance of Roos test, before and after surgical removal of her first rib. The tachycardia resulting from the upper limb manoeuvre was resolved after the surgery leading the authors to assume that the stellate ganglion or the postganglionic efferent sympathetic fibers forming the cardiac plexus were compressed while the Roos test was being performed. However, this comparison is not straightforward. Before surgery, this patient potentially had a compression of the stellar ganglion, but certainly experienced pain. This tachycardia may come from nerve compression, but could also arise from the pain, or other causes. Thus, our study proposes a new assumption regarding the origin of HR increases in TOS patients, as evidenced by the significant HR elevation observed in patients with venous entrapments with an objective comparison.

The diagnosis of TOS remains complex due to varying definitions and ongoing debate over classification systems (Hooper et al., 2010). Diagnosis is generally based on clinical signs and non‐invasive vascular assessments, though the precise prevalence and categorization of TOS subtypes remain subjects of significant discussion in the literature (Maślanka et al., 2023). For example, in this study, strict criteria were applied to classify and include specific patient subgroups. Subjects with isolated venous compression without arterial positional compression were identified based on defined thresholds: a maintained arterial pulsatility amplitude at rest exceeding 25% and a maximum venous emptying of less than 70%.

In this context, the findings of this study could serve as an additional clinical element to assist in more accurately determining the patient's TOS form.

Furthermore, the ‘Pra’ and ‘End’ phases have a significant difference compared to the ‘Rest’ phase for both groups. This increase could result from the activation of the sympathetic nervous system, potentially triggered by discomfort or pain experienced during the manoeuvre. Despite the absence of significant differences between groups for ‘Pra’ and ‘End’ phases (Table 2), we could see a higher median of normalized‐HR values in the last seconds of ‘End’ phase in *V*‐group compared to *C*‐group (respectively, +2 vs. +7 bpm, Figure 2). A longer recording period would have provided a more comprehensive understanding of the cardiovascular disturbances observed in the *V* group, including the return to pre‐mobilization heart rate value, which appears to take longer in patients suffering venous entrapment.

The statistical choices made in this study were aimed at providing robust and interpretable results from repeated physiological measurements. Median values and interquartile ranges were used to summarize HR because they are less sensitive to outliers and skewed distributions, which are common in dynamic HR recordings. EMMs from the linear mixed model were then employed to compare groups and manoeuvre phases, as they allow adjustment for individual variability and the hierarchical structure of repeated measurements. HR values were normalized to each participant's median resting HR to facilitate comparison of changes over time between groups, while minimizing inter‐individual differences in baseline HR. Together, these approaches ensured that the reported effects accurately reflect true group and phase differences rather than being influenced by extreme values or measurement variability (Lenth et al., 2024).

This study is subject to several limitations that should be considered when interpreting the results. First, HR extractions using AcqKnowledge® occasionally presented artifacts that required adjustment due to hardware aberrations or missing data. Consequently, some values had to be reconstructed based on surrounding data points. While efforts were made to ensure accuracy, the possibility remains that these recreated values may not precisely reflect the time‐real physiological responses at those specific moments.

Second, HR measurements were limited to the ‘Su‐Pra’ derivative of the Roos manoeuvre, and no direct evaluation of pain or discomfort was performed. Pain is a known confounding factor, as it can independently trigger sympathetic activation and increase HR. In our study and also in Kaymak et al. (2004) and Özçakar et al. (2008) studies, all participants exhibited symptoms suspected to originate from TOS, which may have influenced discomfort levels and subsequent HR changes (pain was shown to significantly decrease the mean RR interval compared to rest in a study by Terkelsen et al. (2005)). The use of a visual analogue scale or questionnaire to measure perceived pain on multiple occasions during the manoeuvres may provide insight into this issue such as the scale Modified Somatic Perceptions Questionnaire (MSPQ), used in the study of Gockel et al. (1995). However, all participants in this study exhibited symptoms suspected to originate from TOS, meaning that any pain‐related HR increase would likely occur in both groups. Therefore, while pain may contribute to overall HR elevation, it is unlikely to account for the differences observed between groups.

Third, distinguishing the effects of venous compression from those of pain would ideally require testing in individuals without TOC symptoms. Previous research by Raaf (1955), Gilroy & Meyer (1963) and Chen et al. (2014) has indicated that vascular compression occurs in a substantial percentage of asymptomatic individuals (up to 60%). It is well‐documented that a significant proportion of the population may have a thoracic outlet compression without experiencing symptoms. Therefore, a study of a potential HR increase in this asymptomatic population could be more appropriately designed and would remove the pain aspect.

Fourth, the study was not designed to have sufficient statistical power to detect a given difference of interest in each phase. Consequently, it is difficult to ascertain whether the absence of a difference in certain phases suggests an absence of difference in the population or simply reflects a lack of statistical power. Nevertheless, the study is significant as it provides results on which future studies can build to better anticipate the expected effects of the manoeuvre tested.

Lastly, this study focused exclusively on the ‘Su‐Pra’ derivative of the Roos manoeuvre. The selection of this specific manoeuvre was intentional, given its reported efficacy in yielding positive results compared to other tests (Nord et al., 2008; Rayan & Jensen, 1995), despite other diagnostic tests being available for TOS assessment. It has the advantage of highlighting the results of two independent, validated and reliable tools simultaneously. This really limits the possible bias during manoeuvres.

Despite these limitations, the study provides valuable information on HR dynamics during upper limb manoeuvres in venous TOS patients, laying the groundwork for future research to more comprehensively evaluate cardiovascular responses and refine clinical assessment strategies.

## 5 CONCLUSION

This study provides valuable insights into HR changes during dynamic manoeuvres in patients with isolated venous, but not arterial, flow impairment due to thoracic outlet compression, compared to those without any flow impairment.

HR analysis during the ‘Su‐Pra’ manoeuvre revealed distinct cardiovascular responses: patients with venous outflow impairment showed a marked HR increase during compression compared to the *C*‐group, followed by stabilization. Additionally, hemodynamic variations occurred in all manoeuvre phases compared to rest.

These findings enhance the understanding of cardiovascular characteristics and responses in this population, highlighting the complexities in diagnosing and managing this challenging pathology.

## AUTHOR CONTRIBUTIONS

**Quentin Petit**: Software; validation; formal analysis; data curation; writing—original draft; writing—review and editing; visualization. **Simon Lecoq**: Conceptualization; methodology; validation; investigation; writing—review and editing. **Florian Congnard**: Validation; formal analysis; writing—original draft; writing—review and editing; visualization. **Nathan Cronier**: Software; validation; formal analysis; writing—review and editing. **Pierre‐Yves de Müllenheim**: Software; validation; formal analysis; data curation; writing—review and editing; visualization. **Pierre Abraham**: Conceptualization; methodology; validation; formal analysis; investigation; writing—original draft; writing—review and editing; visualization. **Bénédicte Noury‐Desvaux**: Conceptualization; methodology; validation; formal analysis; writing—original draft; writing—review and editing; visualization.

## CONFLICT OF INTEREST STATEMENT

The authors declare no conflicts of interest.

## SOFTWARE INFORMATION

Data analyses were performed using R programming language (version 4.4.1) and the RStudio environment (version 2024.12.0+467) with *dplyr* package (Wickham et al., 2023), *effectsize* package (Ben‐Shachar et al., 2020), *emmeans* package (Lenth et al., 2024), *ggplot2* package (Wickham et al., 2016), *ggrain* package (Allen et al., 2019), *questionr* package (Barnier et al., 2023), *rlang* package (Henry et al., 2025), *skimr* package (Waring et al., 2022), *table1* package (Rich, 2023) and *tidyverse* package (Wickham et al., 2019).

## Data Availability

Data will be made available upon reasonable request to the authors.
